# Salivary proteomics and metaproteomics identifies distinct molecular and taxonomic signatures of type-2 diabetes

**DOI:** 10.1186/s40168-024-01997-5

**Published:** 2025-01-10

**Authors:** Diana Samodova, Evelina Stankevic, Mathilde Sonne Søndergaard, Naiyu Hu, Tarunveer S. Ahluwalia, Daniel R. Witte, Daniel Belstrøm, Anniek Frederike Lubberding, Pratik D. Jagtap, Torben Hansen, Atul S. Deshmukh

**Affiliations:** 1 Novo Nordisk Foundation Center for Basic Metabolic Research, University of Copenhagen, Blegdamsvej 3B, Copenhagen, 2200 Denmark; 2 Department of Biomedical Sciences, University of Copenhagen, Blegdamsvej 3B, Copenhagen, 2200 Denmark; 3 Steno Diabetes Center Copenhagen, Borgmester Ib Juuls Vej 83, Herlev, 2730 Denmark; 4 Department of Biology, The Bioinformatics Center, University of Copenhagen, Ole Maaløes Vej 5, Copenhagen, 2200 Denmark; 5 Department of Public Health, Aarhus University, Bartholins Allé 2, Building 1260, Aarhus, 8000 Denmark; 6 Steno Diabetes Center Aarhus, Aarhus University Hospital, Palle Juul-Jensens, Boulevard 11, Entrance A, Aarhus, 8200 Denmark; 7 Section for Clinical Oral Microbiology, Department of Odontology, University of Copenhagen, Nørre Allé 20, Copenhagen, 2200 Denmark; 8 Department of Biochemistry, Molecular Biology, and Biophysics, University of Minnesota, 420 Washington Ave SE, Minneapolis, MN 55455 USA

**Keywords:** Metaproteomics, Microbiome, Saliva, Type-2 diabetes, DIA-PASEF, Genus, Protein, Peptide, Biomarker

## Abstract

**Background:**

Saliva is a protein-rich body fluid for noninvasive discovery of biomolecules, containing both human and microbial components, associated with various chronic diseases. Type-2 diabetes (T2D) imposes a significant health and socio-economic burden. Prior research on T2D salivary microbiome utilized methods such as metagenomics, metatranscriptomics, 16S rRNA sequencing, and low-throughput proteomics.

**Results:**

We conducted ultrafast, in-depth MS-based proteomic and metaproteomic profiling of saliva from 15 newly diagnosed T2D individuals and 15 age-/BMI-matched healthy controls (HC). Using state-of-the-art proteomics, over 4500 human and bacterial proteins were identified in a single 21-min run. Bioinformatic analysis revealed host signatures of altered immune-, lipid-, and glucose-metabolism regulatory systems, increased oxidative stress, and possible precancerous changes in T2D saliva. Abundance of peptides for bacterial genera such as *Neisseria* and *Corynebacterium* were altered showing biomarker potential, offering insights into disease pathophysiology and microbial applications for T2D management.

**Conclusions:**

This study presents a comprehensive mapping of salivary proteins and microbial communities, serving as a foundational resource for enhancing understanding of T2D pathophysiology. The identified biomarkers hold promise for advancing diagnostics and therapeutic approaches in T2D and its associated long-term complication

Video Abstract

**Supplementary Information:**

The online version contains supplementary material available at 10.1186/s40168-024-01997-5.

## Background

Homeostasis of the oral cavity is maintained by saliva, protein composition of which can differentiate between healthy and diseased individuals [1]. Saliva is an attractive sample for the diagnosis of disease, as it is easily and noninvasively collected, convenient to store, and demands fewer processing steps during clinical procedures, in comparison to blood [2, 3]. Saliva carries biomolecular signatures of both human and microbial origin with potential disease-specific imprints, thus serving as a valuable biomarker source in clinical diagnostics [4]. The human genes and proteins, differentially regulated in saliva of healthy and diseased individuals, offer insights into the immune and metabolic status of the patients [5], while the extremely rich salivary microbiota (bacteria, archaea, fungi, protozoa, and viruses [6]) mediates various physiological functions within the human body [1, 7]. Hence, identification of differences in salivary composition between healthy and diseased individuals, potentially, allows for better understanding of the pathophysiology of systemic diseases.

Type-2 diabetes (T2D) is one of the most prevalent chronic diseases and a major socio-economic burden. T2D is often linked to chronic low-grade inflammation, triggering activation of immune cells that in turn release pro-inflammatory molecules, thereby contributing to progression and complication of the disease [8]. In recent years, there has been a growing interest in examining the salivary composition of individuals with T2D, including both human and bacterial elements [9, 10]. Traditionally, the detection of microbial features in the saliva of T2D individuals mainly relied on methods, such as metagenomics [9, 11], metatranscriptomics [12], and 16S rRNA sequencing [13–16], mostly reporting relative abundance of bacterial genera and phyla in T2D and healthy controls. While providing general information on size and diversity of microbial populations [17, 18], some of these methods offer limited knowledge about microbial functions, local environmental factors [19, 20], and differences in abundance of host features.

Contrastingly, mass-spectrometry (MS)-based metaproteomics and proteomics offer information about protein expression function of the microbiome, as well as specific microorganisms [4, 21, 22], along with taxonomic composition of the microbiome and host protein characterization. Despite this, the metaproteome of T2D saliva remains unexplored. Morever, MS-based proteomics has been previously employed to profile human proteins in T2D saliva [5, 23–27]. However, these pioneering studies were constrained by limited proteome coverage, primarily focusing on highly abundant proteins involved in inflammation and metabolism.

Recent advances in sensitive mass spectrometers [28], innovative data acquisition methods [29], the utilization of reproducible liquid chromatography [30], and integration of powerful computational tools [31, 32] have collectively improved throughput, sensitivity, and coverage of the proteome. We reasoned that these remarkable developments would offer significant improvements in simultaneous quantification of T2D salivary metaproteome, alongside detection of human proteins. We rationalized that saliva, a non-invasive biofluid, provides a unique opportunity to identify novel biomarkers that reflect both host and microbial changes in T2D, potentially complementing or enhancing existing diagnostics. Capitalizing on these advancements, we performed comprehensive proteomics and metaproteomics analysis of saliva from healthy and T2D individuals in a single 21-min MS run. Our findings revealed protein signatures indicating altered host immune-, lipid-, and glucose-metabolism regulatory systems, as well as increased oxidative stress and potential precancerous states in T2D saliva. Furthermore, metaproteomics analysis revealed alteration in abundance of bacterial genera *Neisseria* and *Corynebacterium* in T2D individuals. Our rapid and in-depth profiling of T2D saliva by means of proteomics and metaproteomics both enhances understanding of the disease’s pathophysiology and holds promise for biomarker discovery through extensive profiling of clinical patient cohorts.

## Methods

### Study design

The study participants were selected from the larger ADDITION-PRO cohort (*n* = 2082), which was conducted between 2009 and 2011 [33], in four centers across Denmark. ADDITION-PRO is a risk-stratified cohort, set up to understand the risks and underlying mechanisms that drive progression to T2D and cardiovascular disease in people with pre-diabetes and high diabetes risk identified in a screening program [33]. ADDITION-PRO is nested in the Danish arm of the ADDITION-Europe screening program and trial. The ADDITION trial (2001–2006) evaluated the effect of comprehensive multifactorial target-driven cardiometabolic treatment in general practices on the incidence of cardiovascular events in individuals with screen-detected diabetes [34]. Recruitment for the Danish arm of the ADDITION trial consisted of a population-based screening program set in 181 general practices, with a target population of over 150,000 individuals. Besides identifying people with screen-detected diabetes, the screening program uncovered a large group of individuals in various pre-diabetic stages or at high risk for diabetes [35]. These individuals were then selected as the participant pool for the ADDITION-PRO study in 2009–2011. The study was conducted in accordance with the principles of the Declaration of Helsinki.

Participants have undergone an extensive clinical examination including anthropometrics, detailed characterization of glycemic status, which has been reported in detail elsewhere [33]. In total, 15 patients with screen-detected T2D and 15 individuals with normoglycemia (termed as HC) from the ADDITION-PRO cohort were selected for this study. The groups were selected to approximately match in age, gender, and BMI.

### Sample collection

A detailed description of saliva sample collection is provided elsewhere [36]. Briefly, saliva samples have been collected at Steno Diabetes Center Copenhagen, Copenhagen, Denmark, in 2009–2011. Participants were asked not to brush their teeth on the day of sample collection. Saliva production was stimulated by chewing paraffin wax gum, and collected samples were stored immediately at − 80 °C and kept un-thawed until analysis in 2022.

### Sample preparation

Saliva sample preparation was performed by boiling 20 µL of saliva (10 min, 99 °C) with 10 µL of the lysis buffer (1% SDC/100-mM Tris, pH 8.5/10-mM TCEP/40-mM CAA) to denature proteins, followed by 4 min sonication. Approximately, 30 µg of protein was digested with 1:250 enzyme-to-protein ratio of Lys-C (Sigma-Aldrich) for 1 h at 37 °C, followed by an overnight digestion with 1:100 enzyme-to-protein ratio of Trypsin (Sigma-Aldrich), at 37 °C. Digestion reaction was quenched by 1:10 acidification with 10% TFA (Sigma-Aldrich, T6508-500ML). All samples were spun down for 5 min at 17,000 × *g*, and peptide concentration was determined with a NanoDrop spectrophotometer (Thermo, Wilmington, DE, USA), followed by purification on an in-house packed StageTip C_18_ cartridges with further loading of ~ 200 ng of material on Evosep C_18_ cartridges (Evotips) (Evosep Biosystems, Denmark). Evotips were washed with 35% ACN, and peptides were loaded directly on the Evosep One [37] LC system (Evosep Biosystems, Denmark) for further separation and analysis.

### LC–MS/MS analysis

Peptides were analyzed using Evosep One LC (Evosep Biosystems, Denmark) coupled to timsTOF Pro 2 mass spectrometer (Bruker, Bremen, Germany) [29]. Peptides were separated on a 8-cm analytical PepSep column (Bruker Daltonik, Germany), 150-µm inner diameter, packed with 1.5-µm C_18_ material, using a 21-min long preset LC gradient method (60 samples per day), and injected via a CaptiveSpray source and 10-μm emitter into a timsTOF Pro 2 mass spectrometer. The mass spectrometer was operated in positive ion mode, using DIA-PASEF acquisition [38]. Briefly, the DIA-PASEF scan range was set to 400–1200 (m/z), the TIMS mobility range to 0.6–1.43 (V cm^−2^), and ramp and accumulation times to 100 ms. Thirty-two windows of 25-Da width each were placed in the m-/z-ion mobility plane. The diaPASEF scheme was set to 2 repetitions of 16-scan. The resulting estimated cycle time was 1.80 s.

### Saliva sample processing and 16S rRNA gene amplicon sequence processing

DNA extraction of saliva samples, 16S rRNA gene amplicon sequencing, and raw sequence data processing were done as reported [36]. Briefly, microbial DNA was isolated using the NucleoSpin Soil kit, with bacterial cells lysed using SL1 + Enhancer Buffer SX. DNA quality and quantity were assessed with a Qubit 2.0 fluorometer and a NanoDrop 2000 spectrometer. Genomic DNA was standardized to 30 ng for PCR amplification of the 16S rDNA V4 region, with PCR products purified using the AMPure XP kit. Library quality was assessed using a 2100 bioanalyzer and qPCR with EvaGreen™.

Raw 16S rRNA gene sequencing data processing was done using dada2 [39] and metabaRpipe. This included truncation, filtering, error rate learning, and ASV inference using a pseudo-pooling strategy. Taxonomic classification used dada2 and the eHOMD database (version V15.22, https://www.homd.org), and a phylogenetic tree was constructed using the phyloseq package v1.22.3 [40].

### Raw LC–MS/MS data processing

DIA raw MS data were analyzed with Spectronaut [32] v17 using an in-house generated sample-specific protein fasta file, containing 1,068,210 protein sequences (reviewed *Homo sapiens* UniProt/Swiss-Prot proteome (proteome ID: UP000005640, downloaded 2021 June 25) + individual-specific protein sequences from Human Oral Microbiome Database (HOMD) [41]), in directDIA mode. The sample-specific fasta was generated, using Linux command [42] application and RStudio [43], as follows: (i) 16S rRNA sequencing data from the corresponding study individuals were first combined into a single fasta file, followed by removing duplicate entries, and (ii) the merged 16S rRNA fasta was then aligned to the 16S rRNA RefSeq sequences from HOMD (https://www.homd.org/), using local BLASTn [44] tool executed via Linux command application. For each sequence, only the most homologous alignments were retained (top 200 matches with the alignment score > 450); (iii) finally, all corresponding reference protein IDs (HMT-id) were extracted from HOMD for each of the 16S rRNA sequences, thus forming a reduced sample-specific reference database of bacterial proteins.

The default settings in Spectronaut were used, unless otherwise noted. Data filtering was set to “Qvalue.” False discovery rate (FDR) was set to 1% at peptide precursor level and 1% at protein level. Proteotypic-only peptides were used for protein quantification.

### Bioinformatics analysis

Data analysis was performed using Perseus software [45] v1.6.14.0 and R programming language within the RStudio v 4.1.2 environment, utilizing in-house developed R [43] scripts. Peptide-, protein-, and genus-level analysis was carried out.

To conduct protein-level analysis, the raw proteome data was preprocessed by log2 transformation of the intensity values, followed by omitting variables with less than 70% present values, and the resulting missing values were imputed by drawing random samples from a normal distribution with downshifted mean by 1.8 standard deviation (SD) and scaled SD (0.3) relative to that of abundance distribution of the corresponding protein across all samples. For multivariate analysis, a principal component analysis was performed on the processed data. The percentage of contribution of each of the principal components was determined with factoextra [46] package, while drivers of principal component separation were determined, using FactoMineR [46] package. The first and second principal component was used for sample projections. Univariate analysis was performed using two-sample *t*-test (Benjamini–Hochberg *FDR* < 0.05), and functional annotations of the proteins were obtained from the following: (i) UniProt for human entries and (ii) eggNOG-mapper [47] v5 (http://eggnog-mapper.embl.de/) for bacterial proteins respectively. BLASTp NCBI functionality (https://blast.ncbi.nlm.nih.gov/Blast.cgi?PROGRAM=blastp&PAGE_TYPE=BlastSearch&LINK_LOC=blasthome) was used to assess specificity of bacterial peptides [48]. 1D annotation enrichment analysis [49] of differentially expressed proteins was performed on both human and bacterial protein intensity log2-fold changes, calculated between T2D and HC groups (Benjamini–Hochberg *FDR* < 0.02) and visualized as a bubble plot, using bubbleHeatmap [50] package. For peptide- and genus-level analysis, unique bacterial peptides were preprocessed with Unipept Desktop [51] v2.0.0 software to (i) assign corresponding taxonomic levels, using the lower common ancestor (lca) approach [52], and (ii) to perform functional annotation of bacterial peptides, using annotations from entire UniProt database. Relative abundance of bacterial genera and phyla was determined by calculating the summed peptide/16S rRNA read intensity per genus/phylum, followed by dividing with the total intensity, for both metaproteomics and 16S rRNA datasets. Statistical significance of the differences in relative abundance of bacterial genera between T2D and HC groups was assessed using a two-sample *t*-test (Benjamini–Hochberg *FDR* < 0.05). Pearson correlation coefficient value (*P* < 0.01) was calculated, using the total intensities of all bacterial genera, to assess the correlation between the metaproteomics and 16S rRNA datasets. To perform statistical and functional enrichment analysis on the peptide level, peptide intensity values were log2-transformed, followed by omitting variables with less than 70% present values, and the resulting missing values were imputed by random forest method with the missForest package. Human peptides were then removed from the dataset, followed by the three separate types of analysis for bacterial peptides: (i) back-transformation of the log2-transformed peptide intensity values, followed by calculation of the summed peptide intensity per genus (only unique genus-specific bacterial peptides were considered), log2-transformation of the total genus intensity, and univariate analysis of differentially expressed bacterial genera using a two-sample *t*-test (Benjamini–Hochberg *FDR* < 0.05); (ii) 2D annotation enrichment analysis (Benjamini–Hochberg *FDR* < 0.02) [49] of all bacterial peptides between the two groups (T2D vs HC), visualized as a scatter plot (functional annotations extracted from Unipept); and (iii) unsupervised hierarchical clustering (Euclidean distance metric) of bacterial peptides from genera *Neisseria* and *Corynebacterium*, followed by functional enrichment analysis (functional annotations extracted from Unipept), performing Fisher’s exact test (Benjamini–Hochberg *FDR* < 0.02) on each cluster. For univariate analysis of bacterial genera, derived from the 16S rRNA reads, similar filtration and imputation steps were carried out, as for the peptides.

A linear regression model was applied to test the associations between bacterial peptides from genera *Neisseria*, *Corynebacterium*, and *Haemophilus* and corresponding clinical variables, where peptide log2 abundance was an independent variable and clinical trait was a dependent variable, all while controlling for relevant covariates (age, sex, smoking, and glycemic status). We fitted an interaction regression model to estimate if glycemic status (HC versus T2D) modifies the associations between bacterial peptides and corresponding clinical variables. Prior to the analysis, the dependent variables were log-transformed and scaled. The results were computed using the “stats” package in R and thereafter reported as effect size estimates on log2 scale and FDR adjusted [53] *P*-values. *P*-values were considered significant below 0.05 after FDR correction of 5% and suggestive below 0.05 before FDR adjustment.

### Validation of bacterial peptides using synthetic peptide hybrid library

Top7 peptide sequences from bacterial genera *Corynebacterium* and *Neisseria*, with the highest quality of the annotated MS/MS spectra and the highest MS score (EG.Cscore, Spectronaut), were selected and synthesized by JPT Peptide Technologies GmbH (Berlin, Germany). Synthetic peptide hybrid library was created, as described before [54]. In brief, synthetic peptides were dissolved in 0.1% FA and pooled in equimolar concentrations, to obtain a stock solution. Synthetic peptides were then spiked into 0.5-μg tryptic peptides from yeast lysate, serving as a background, at five different ratios (1:1, 1:2, 1:4, 1:10, and 1:20). These mixtures were loaded on C18-based Evotips and analyzed with the same LC–MS/MS DIA method, as the study samples, in triplicates. Raw MS data were searched with Pulsar functionality within Spectronaut v18 software, to generate hybrid spectral library using fasta file composed of *Saccharomyces cerevisiae* (UniProtKB/Swiss-Prot database, downloaded August 2023) and corresponding synthetic peptide sequences. Subsequently, DIA MS raw files from the study samples were reanalyzed, using the abovementioned hybrid spectral library, with Spectronaut v18, to validate the presence/quantify peptides of interest.

### Functional peptide validation by insulin secretion assay

#### Cell culture

The rat INS-1 insulinoma cell line (kindly provided by C. Wollheim and P. Maechler, University of Geneva, Switzerland) was cultured in RPMI-1640 medium with GlutaMAX supplemented with 10% fetal bovine serum (FBS) (v/v), 100 IU/mL penicillin, 100 μg/mL streptomycin, 10 mmol/L HEPES, 50 μmol/L *β*-mercaptoethanol (all Thermo Fisher Scientific, USA), and 1 mmol/L sodium pyruvate (Merck, Denmark) at 37 °C, 5% CO2, humidified.

#### Glucose-stimulated insulin secretion assay

For glucose-stimulated insulin secretion (GSIS), 200,000 INS-1 cells/well were seeded (12-well plate) and preincubated in cell culture medium for 72 h. Subsequently, media was removed, and cells were incubated for 2 h with pre-warmed, fresh Krebs–Ringer’s-bicarbonate-HEPES (KRBH) buffer (in mM: 135 NaCl, 3.6 KCl, 1.5 CaCl2, 1.2 KH2PO4, 1.2 MgSO4, 2 NaHCO3, 20 HEPES; 0.1% BSA, pH 7.4) at 2 mM glucose. Buffer was replaced with new, pre-warmed 2 mM glucose KRBH buffer with or without 1 µM bacterial peptides (eluted in deionized water) or vehicle (equivalent volume of deionized water). After 1 h incubation for baseline insulin secretion, supernatant was collected and placed on ice. This was followed by 1 h incubation at 20 mM glucose with 1 µM bacterial peptides or vehicle and supernatant sample collection. Cells were collected with RIPA (Merck, Denmark) supplemented with Halt™ protease inhibitor cocktail EDTA-free (Thermo Fisher Scientific, USA), and protein concentration was determined for normalization by Micro BCA Protein Assay (Thermo Fisher Scientific, USA) according to manufacturer’s protocol. Secreted insulin in supernatants was measured using a rat insulin ELISA kit (Mercodia, Sweden) according to manufacturer’s protocol with 4 × dilution of high glucose samples.

## Results

### Study population

The study population consisted of 30 individuals from the Danish ADDITION-PRO cohort (2009–2011) [33]. ADDITION-PRO is a risk-stratified cohort, aiming to understand the risks and underlying mechanisms that drive progression to T2D and cardiovascular disease among 2082 Danish adults with prediabetes and high diabetes risk identified in a screening program [33]. In total, 15 participants with screen-detected T2D (based on the 1999 WHO criteria, i.e., based on fasting and 2-h oral glucose tolerance test (OGTT) criteria) and 15 participants with normal glucose tolerance were selected for this study and grouped into T2D and healthy controls (HC), respectively. During the selection, participants were matched on age, sex, and BMI. Participant characteristics can be found in Table 1.

**Table 1:** Baseline study participant characteristics

	**Total**	**HC**	**T2D**	***p*-value**
*n*	30	15	15	
BMI, kg/m^2	28.46 (2.86)	28.24 (2.58)	28.68 (3.18)	0.68
Age, years	66.18 (4.48)	66.25 (4.43)	66.10 (4.68)	0.93
Sex: male, *n* (%)	18 (60.0)	9 (60.0)	9 (60.0)	
Current smokers, *n* (%)	6 (20.0)	2 (13.3)	4 (26.7)	
Ex-smokers, *n* (%)	16 (53.3)	9 (60.0)	7 (46.7)	
Nonsmokers, *n* (%)	8 (26.7)	4 (26.7)	4 (26.7)	
P-Glucose, T0 (mmol/L)	6.31 (0.86)	5.68 (0.23)	6.94 (0.81)	< 0.001
HbA1c, %	5.80 (0.42)	5.62 (0.29)	5.99 (0.45)	0.01

General characteristics of HC and T2D patient groups. The variables are displayed as mean values with + / − standard deviation (SD). Student’s *t*-test was used to compare the two groups. A cutoff of *P* < 0.05 was considered for assessing statistical significance. *BMI*, body mass index; *P-Glucose*, T0, plasma glucose fasting; Age, patient age; *HbA1c*, hemoglobin A1c; male, sex male; female, sex female; current smokers, smoking status (smoker); ex-smokers, smoking status (ex-smoker); nonsmokers, smoking status (nonsmoker)

### Salivary proteomics and metaproteomics

**Fig. 1:**
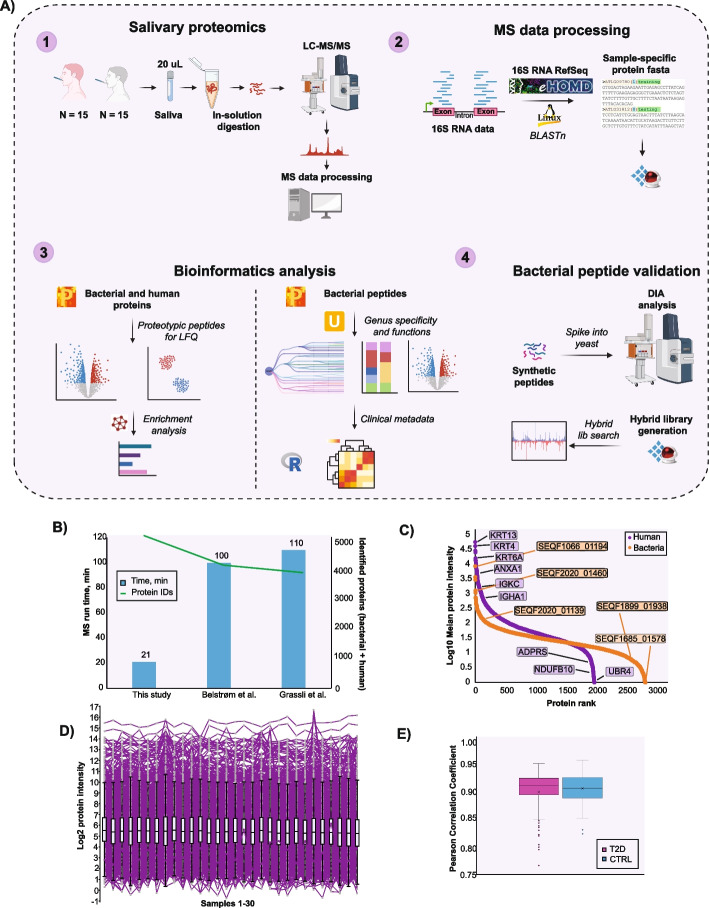
Experimental design and quality control of the study. **A** Study design and experimental setup; **B** Total number of identified proteins; **C** Dynamic range of quantified proteins (LFQ, label-free quantitation values), proteins are ranked according to their abundance (protein rank); **D** Log10 mean protein intensity distribution across all samples; **E** Mean Pearson correlation coefficient of protein intensities in each patient subgroup (*r* > 0.9)

To investigate T2D-associated salivary proteins, we analyzed saliva using an ultrafast LC/MS method involving 21 min measurement time in combination with a new MS acquisition method called DIA-PASEF [55]. We searched the raw MS data in directDIA mode using human reviewed FASTA from UniProt and a sample-specific bacterial protein FASTA. The latter was created by aligning the available 16S rRNA sequencing data from study participants against reference 16S rRNA sequences from HOMD [56]. The in silico generated spectral library consisted of 7787 proteins (2212 protein groups), 15,790 peptides, and 24,385 precursors. The study samples were measured without prior protein depletion, resulting in 30 LC–MS/MS datasets (Fig. 1A).

Using rapid LC–MS/MS analysis, we quantified a total of 4820 (1985 human and 2835 bacterial) proteins (on average 2231 proteins per individual), with MS signals spanning an abundance range of 5 orders of magnitude for human proteins and 4 orders of magnitude for bacteria. This included detection of high-abundant human keratins (KRT13, KRT4, KRT6A), cytoskeletal proteins (ANXA1), immunoglobulins (IGKC, IGHA1), and bacterial ribosomal proteins (SEQF1066_01194, SEQF2020_01460, SEQF2020_01139), as well as low-abundant proteins (UBR4, ADPRS, NDUFB10, SEQF1685_01578, SEQF1899_01938) of both human and bacterial origin (Fig. 1B, C; Additional file 1). We achieved an impressive depth of coverage in both salivary proteome and metaproteome using just 21 min LC–MS runs, a substantial improvement over earlier studies requiring significantly longer measurement time to achieve similar depth of coverage [20, 57] (Fig. 1B). Importantly, our workflow provides an unprecedented profile of salivary metaproteome from individuals with T2D. The data was normally distributed (Fig. 1D), and excellent correlation between biological replicates (median *r*_T2D_ = 0.91; *r*_HC_ = 0.91) was apparent (Fig. 1E), supporting high reproducibility of sample handling and analysis.

### Protein-based profiling of type-2 diabetes saliva

**Fig. 2:**
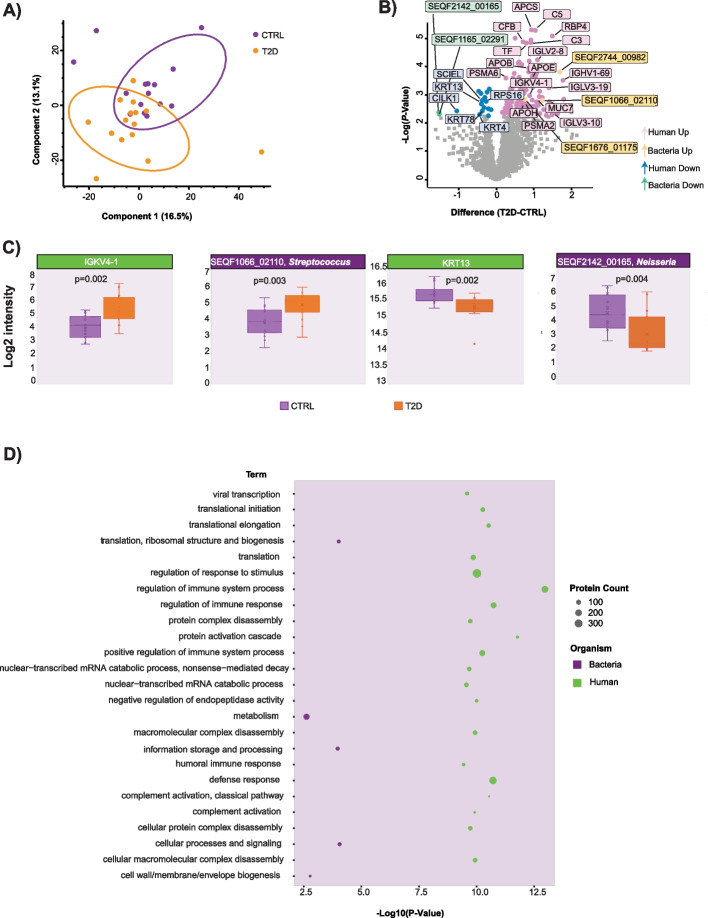
Statistical analysis of salivary proteome and metaproteome. **A** Principal component analysis of salivary proteins from 15 type-2 diabetic individuals and 15 healthy controls; **B** Volcano plot reflecting results of univariate statistical analysis (two-sample t-test) of proteins from the two patient groups. The x-axis displays log2-transformed fold change, while the y-axis displays the -log10-transformed *p*-value. Each point represents one protein, while colours designate regulation and protein classes (bacterial/human); **C** Box-plot visualization of the four selected proteins of interest. Protein expression levels are displayed, as log2-transformed intensity values. A two-sample t-test was used to statistically compare the two groups (HC vs T2D), where *p*-value is shown in the upper portion of the graph. Colors indicate group; **D** Bubble plot visualization of the functional enrichment analysis of differentially-expressed bacterial and human proteins between 15 type-2 diabetic individuals and 15 healthy controls. The x-axis displays the -log10-transformed *p*-value, while size of points represents protein count for each enriched term and color stands for the class (bacteria/human)

When comparing fasting saliva proteome profiles of T2D patients (15 individuals) and HC (15 individuals), a moderate separation of the 2 groups was observed in principal component analysis (PCA) plot (Fig. 2A). Components 1 (PC1) and 2 (PC2) together explained 29.6% of the variation (PC1 16.5%, PC2 13.1%, Fig. 2A; Supplementary Fig. 1A), which was primarily driven by human proteins with enzymatic activity (PTPRJ, PYGL, PTPRC, CAP1, LTA4H etc.), proteins related to immune response (ITGAM, CD14, LGALS3BP), calcium-binding proteins (S100P, S100A4, CALU), actin (ACTN1), and actin-binding proteins (PFN1, GSN, MSN) (Supplementary Fig. 1B). Statistical analysis returned 148 significantly differentially abundant proteins between T2D and HC groups (Student’s *t*-test, *P* < 0.05, Benjamini–Hochberg *FDR* < 0.05), of which 131 proteins (128 human, 3 bacterial) were upregulated and 17 (15 human, 2 bacterial) were downregulated in T2D condition (Fig. 2B; Additional file 2).

Predominantly up-regulated human proteins in the T2D group were associated with immune system regulation and inflammation including immunoglobulins (IGHV3-64, IGHV1-69, IGHV3-43, IGLV3-19, IGHG3, IGKV4-1, IGLV1-51, IGLV2-8; Fig. 2B) complement components (C3, C5, CFH, CFB) as well as acute-phase proteins (APCS, A2M, CP). Interestingly, approximately 40% of up-regulated immunoglobulin proteins comprised immunoglobulin light chains kappa (*κ*) and lambda (*λ*) suggesting association with autoimmune disease or cancer [58–60]. Human proteins associated with lipid metabolism (APOE, APOB, APOA4) and proteasome subunit proteins (PSMA2, PSMA3, PSMA4, PSMA6, PSMB1, PSME2) were also upregulated. Additionally, protein RBP4, a well-known adipokine associated with insulin resistance in T2D [61], and mucin (MUC7) [62] were upregulated, serving as quality controls for our analysis. Although we identified a higher number of bacterial proteins, compared to the human, minimal regulation was observed for these entries in T2D saliva, in contrast to the pronounced changes in abundance of the human proteins. The saliva from T2D individuals had higher expression of putative bacterial proteins from genera *Eubacterium* (SEQF2744_00982), *Streptococcus* (SEQF1066_02110), and bacterial membrane lipoprotein TmpC from genus *Parvimonas* (SEQF1676_01175) (Fig. 2B and C). The identity and taxonomic assignment of the abovementioned bacterial proteins were thereafter verified using BLASTp NCBI functionality. The BLASTp-based protein assignment for SEQF2744_00982 and SEQF1676_01175 differed as compared to Unipept analysis, presumably due to differences in underlying databases. However, it confirmed a 100% match of the protein SEQF1066_02110 to the amylase-binding protein AbpA from genus *Streptococcus* (Fig. 2C), which is known to facilitate catabolism of dietary starches [63].

On the contrary, only a few down-regulated human and bacterial proteins were observed in the T2D group. This included significant downregulation of several keratins (KRT78, KRT13, KRT4; Fig. 2B), keratinocyte sciellin (SCEL), ribosomal protein S16 (RPS16), cytoskeletal proteins (SH3GLB2, SPTAN1, ANXA2), and several proteins with enzymatic activity (CILK1, PPP2R1A, NAGK). Downregulation of bacterial proteins included Efem/EfeO family lipoprotein (SEQF1165_02291) and copper-containing nitrite reductase (SEQF2142_00165) from genus *Neisseria* (Fig. 2B and C). The BLASTp-based verification procedure for the down-regulated bacterial proteins was performed similarly, as for the up-regulated bacterial proteins. The results showed a 100% match for the corresponding *Neisseria* proteins in both cases (Fig. 2C).

To gain deeper insights into differentially regulated human and bacterial proteins in the saliva samples from T2D individuals, we performed a 1D [49] functional enrichment analysis (Additional file 3). The resulting enrichment bubble plots, summarizing the top 20 human biological processes and significantly enriched bacterial terms, are displayed in Fig. 2D. We observed multiple significantly enriched immunity-related human biological processes, such as regulation of immune system process, defense response, regulation of immune response, positive regulation of immune system process, and indicative of immune system activation in T2D patients (Fig. 2D, Additional file 3). Microbiologically, significantly enriched biological processes included metabolism, cell signaling, and biogenesis (Fig. 2D, Additional file 3). Additionally, translation-associated biological processes were significantly enriched for both human and bacterial proteins (Fig. 2D, Additional file 3).

### Taxonomic signature of type-2-diabetic salivary microbiota

To perform an in-depth comparison of the salivary microbiota from HC and T2D individuals, we assigned identified bacterial peptides to the specific taxonomic levels, using the lowest common ancestor (lca) approach [52]. Of the 7452 bacterial peptides, 47% could be assigned to the genus level and 69% to the phylum level, respectively (Table 2).

**Table 2:** Overview of metaproteomics identifications

Unique identified peptides, total	
Human	11,548
Bacterial	7452
Mapped to genus level (%)	3519 (47)
Mapped to phylum level (%)	3150 (69)

**Fig. 3:**
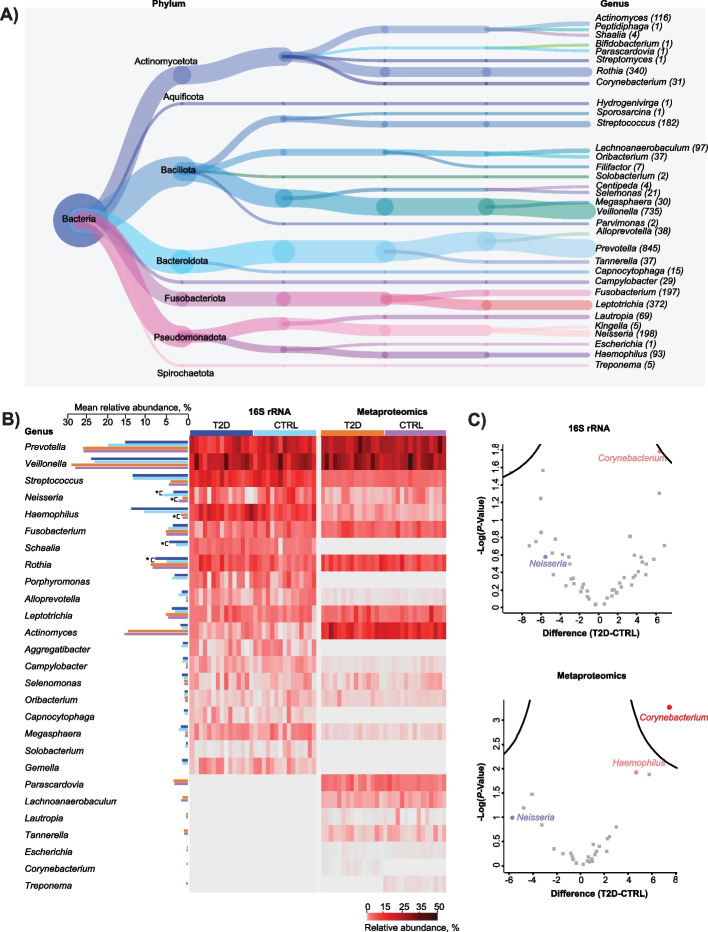
Taxonomic signature of type-2 diabetic microbiome. **A** Taxonomic tree depicting bacterial genera and phyla, identified in the study samples. Values in parenthesis indicate the number of unique bacterial peptides assigned to a particular genus; **B** Relative abundance of the top20 bacterial genera in metaproteomics and 16S rRNA datasets. The abundance values are visualised in a heatmap, while mean relative abundances are plotted as bar plots. Statistical significance is assessed using a two-sample t-test and significant genera are labelled with a *. Colors indicate groups (T2D vs HC); **C** Volcano plots reflecting results of univariate statistical analysis (two-sample t-test) of bacterial genera from the two patient groups, within metaproteomics and 16S rRNA datasets. The x-axis displays log2-transformed fold change, while the y-axis displays the log10-transformed *p*-value. Each point and color represents one bacterial genus

This approach led to identification of a total of 33 bacterial genera and 7 phyla (Fig. 3A).

We leveraged existing 16S rRNA sequencing data and compared the relative abundance of the 20 most abundant bacterial genera between 16S rRNA and metaproteomics datasets (Fig. 3B). The comparison revealed several differences in the composition of the predominant bacterial genera across the two datasets. For instance, in the metaproteomics dataset, the top 5 most abundant bacterial genera were *Prevotella*, *Veillonella*, *Actinomyces*, *Rothia*, and *Fusobacterium*, while 16S rRNA data displayed *Veillonella*, *Prevotella*, *Haemophilus*, *Streptococcus*, and *Rothia* as the most prevalent genera (Fig. 3B). These genera collectively represented approximately 70% of taxonomic abundance within corresponding datasets.

Furthermore, we performed a statistical analysis on the relative abundance of the top 20 bacterial genera from the two datasets. In the 16S rRNA dataset, we observed significantly higher relative abundance (Student’s *t*-test, *P* < = 0.05, Benjamini–Hochberg *FDR* < 0.05) of genera *Rothia* (*P* = 0.05) and *Schaalia* (*P* = 0.01) in T2D saliva, compared to HC group. Conversely, we found a significantly lower relative abundance of the genus *Neisseria* (*P* = 0.05). In the metaproteomics dataset, we observed significantly higher relative abundance of genus *Haemophilus* (*P* = 0.02) and significantly lower relative abundance of genus *Neisseria* (*P* = 0.05) in the T2D group, compared to HC. Additionally, several complementary bacterial genera were identified within the top 20 entries across the 2 datasets. Genera *Schaalia*, *Porphyromonas*, *Aggregatibacter*, *Capnocytophaga*, *Solobacterium*, and *Gemella* were exclusively present within the top 20 entries of the 16S rRNA dataset, while *Parascardovia*, *Lachnoanaerobaculum*, *Lautropia*, *Tannerella*, *Escherichia*, *Corynebacterium*, and *Treponema* were exclusively identified among top 20 metaproteomics genera. Importantly, genus *Corynebacterium* was only identified in the T2D group while genus *Treponema* only in the HC group. Despite differences in the abudance of most prevalent genera (Fig. 3B), the relative abundance of the top 20 bacterial genera in two datasets showed high degree of correlation (Pearson correlation coefficient = 0.7164; *P* < 0.01) (Supplementary Fig. 2). A full list of all bacterial genera and phyla, identified with the two methodologies, is presented in Additional file 4. In light of taxonomic differences between the two datasets, we believe in reliability of metaproteomics analysis, since the abovementioned findings were validated by a hybrid MS analysis of synthetic peptides (Fig. 5B; Supplementary Fig. 4).

When performing statistical comparison of the two datasets on the total log2-transformed intensities of bacterial genera (Student’s *t*-test, *P* < 0.05, Benjamini–Hochberg *FDR* < 0.05), we observed *Corynebacterium* as the only significantly up-regulated bacterial genus in T2D saliva (Fig. 3C), compared to healthy controls, within the metaproteomics dataset, while there was no significant regulation in the 16S rRNA data.

### In-depth functional assessment in type-2 diabetes salivary microbiota

**Fig. 4:**
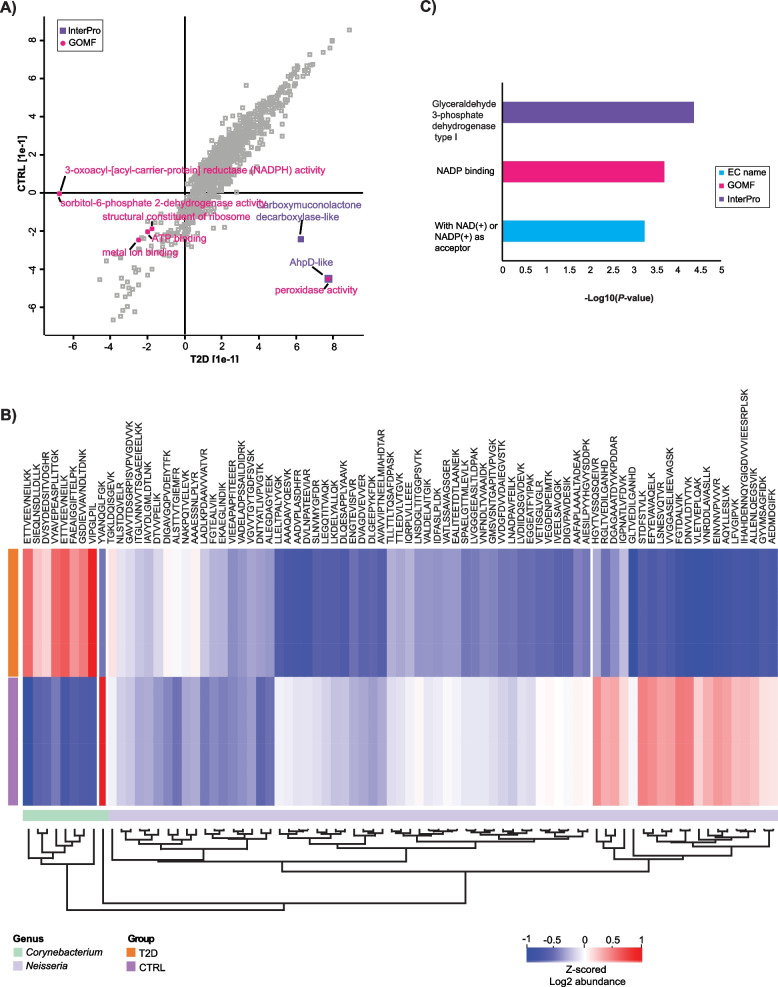
Bacterial functions in type-2 diabetes oral microbiome. **A** Scatter plot visualization of the functional enrichment analysis of all bacterial peptides between the two groups (T2D vs HC). The x- and y-axis display enrichment factor for each functional term, within each patient group. Points are colored and annotated according to the corresponding functional terms; **B** Unsupervised hierarchical clustering of bacterial peptides from genera *Neisseria* and *Corynebacterium*, visualized as a heatmap, based on the Z-scored log2-transformed intensities of bacterial peptides. The colors indicate groups (T2D vs HC) and genera (*Neisseria* vs *Corynebacterium*); **C** Bar plot reflecting the top3 significantly enriched functions. Colors correspond to the term class and x-axis displays the -log10-transformed *p*-value

To assess the differences in biological functions of type-2 diabetic oral microbiota, compared to healthy controls, we performed a 2D functional enrichment analysis [49] on all bacterial peptides, in an unbiased manner (Additional file 5). The resulting enrichment scatter plot is displayed in Fig. 4A.

We observe several enzymatic terms, such as peroxidase activity (two peptides, *P* < 0.005)/AhpD-like (two peptides, *P* < 0.005) and carboxymuconolactone decarboxylase-like (three peptides, *P* < 0.005) being positively enriched (*P* < = 0.005, Benjamini–Hochberg *FD*R < 0.02) in the T2D group and negatively enriched in the HC group. Conversely, terms ATP binding (728 peptides, *P* < 0.005), metal ion binding (258 peptides, *P* < 0.005), structural constituent of ribosome (434 peptides, *P* < 0.005), and 3-oxoacyl-[acyl-carrier-protein] reductase (NADPH) activity/sorbitol-6-phosphate 2-dehydrogenase activity (2 peptides, *P* < 0.005) are negatively enriched both in T2D and in control patient groups.

We also performed unsupervised hierarchical clustering (Fig. 4B) on unique bacterial peptides from genera *Neisseria* and *Corynebacterium*, which previously displayed significant differences in abundance between the two patient groups (Fig. 3B and C). Subsequently, we subjected resulting clusters to the enrichment analysis (*Fisher’s* exact test on the cluster; *P* < = 0.005, Benjamini–Hochberg *FDR* < 0.04). This analysis revealed several glycolysis-related terms that were significantly enriched the in cluster, exclusively containing peptides from genus *Corynebacterium*: glyceraldehyde 3-phosphate dehydrogenase type I (four peptides, *P* < 0.005), NADP binding (five peptides, *P* < 0.005), and with NAD( +) or NADP( +) as acceptor (six peptides, *P* < 0.005) (Fig. 4B and C).

### Host–pathogen interactions in type-2 diabetic oral microbiome

**Fig. 5:**
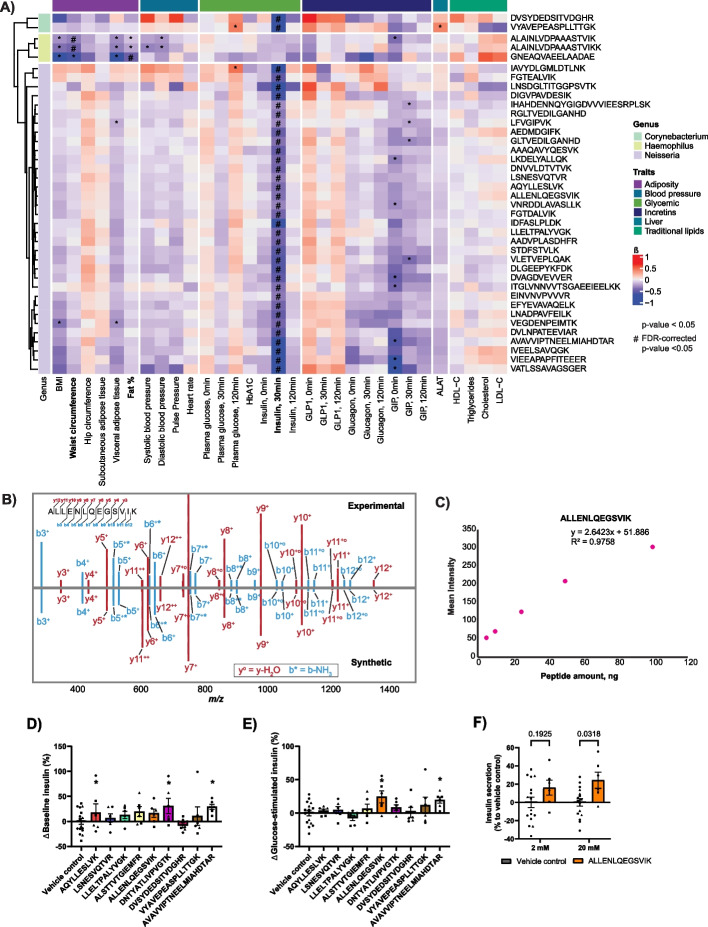
Bioclinical value of bacterial peptides of interest. **A** Clinical association analysis of bacterial peptides from genera *Corynebacterium*, *Haemophilus* and *Neisseria*, arranged as a supervised hierarchical clustering and visualized as a heatmap, based on scaled log2-transformed peptide intensities. Colors indicate specific classes of cardiometabolic traits used in association analysis, as well as bacterial genera. Only significantly-associated peptides are shown; **B** An example of an annotated mirror MS/MS spectrum, used for verification of peptide identity by synthetic peptide hybrid library search; **C** An example calibration curve of synthetic peptide spikes into yeast at the different ratios; **D&E** Screen of nine bacterial peptides on insulin secretion at low glucose (baseline, 2 mM glucose) and high glucose (20 mM) compared to vehicle control in INS-1 β-cell line. Peptides AQYLLESLVK, DNTYATLIVPVGTK and AVAVVIPTNEELMIAHDTAR (genus *Neisseria*) nominally increased insulin secretion at low glucose (*), peptides ALLENLQEGSVIK and AVAVVIPTNEELMIAHDTAR (genus *Neisseria*) nominally increased insulin secretion at high glucose (*). Data was screened with multiple unpaired t-tests and depicted as % difference from control values of individual batches (batches indicated by symbols); **F** Highlight of bacterial peptide ALLENLQEGSVIK from screen in D&E, depicting % difference in insulin secretion at low glucose (2 mM) and high glucose (20 mM) in INS-1 cells in the presence and absence of the bacterial peptide ALLENLQEGSVIK compared to vehicle control. Tested with two-way ANOVA with Sidak’s multiple comparison testing; data depicted as % difference from control values of individual batches (batches indicated by symbols)

We performed association analysis to examine the relationship between bacterial peptides and cardiometabolic traits while adjusting for age, sex, smoking, and glycemic status, which resulted in several positive and negative associations (Fig. 5A; Supplementary Fig. 3A; Additional file 6).

Bacterial peptides, used in the association analysis, originated from the differentially expressed bacterial genus *Corynebacterium* (Fig. 3C), as well as from genera *Neisseria* and *Haemophilus*, which showed statistically significant differences in relative abundance between the two patient groups (Fig. 3B). The resulting association matrix is depicted in Figs. 4 and 5 and in Additional file 6. Interestingly, two peptides from genus *Corynebacterium* and multiple peptides from genus *Neisseria* displayed significant negative association with plasma insulin levels after 30 min of tOGTT (ALLENLQEGSVIK, FDR-corrected *P* < 0.05; LSNESVQTVR, FDR-corrected *P* < 0.05; VYAVEPEASPLLTTGK, FDR-corrected *P* < 0.05, etc.), while genus *Haemophilus* did not follow this pattern (Additional file 6). In addition, two peptides from genus *Haemophilus* displayed significant negative association (ALAINLVDPAAASTVIK and ALAINLVDPAAASTVIKK, FDR-corrected *P* < 0.05) with the patient waist circumference, while one peptide (GNEAQVAEELAADAE, FDR-corrected *P* < 0.05) was significantly negatively associated with fat % (Additional file 6). We subsequently examined whether glycaemic status affects the identified trait-peptide associations through an interaction model. We did not see significant interaction effects on the FDR-significant peptide-trait associations (Additional file 8).

We validated our observations using targeted proteomics. Briefly, we used a hybrid MS library, containing 14 synthetic peptides with the lowest FDR-corrected *p*-values from association analysis (peptides VIPGLPIL, AQYLLESLVK, LSNESVQTVR, LLELTPALYVGK, ALSTTVTGIEMFR, ETTVEEVNEILKK, ALLENLQEGSVIK, FAEAIGGIFTELPK, DNTYATLIVPVGTK, DVSYDEDSITVDGHR, GSDIEVVAVNDLTDNK, VYAVEPEASPLLTTGK, AAFAPLAAALTADEAK, AVAVVIPTNEELMIAHDTAR). We then reanalyzed our data using this library. Employing this approach, we successfully verified the identity and presence of 7 peptides out of 14 (Supplementary Fig. 4), as well as determined their concentrations in patient saliva (Additional file 7). As an illustration, Fig. 5B displays a representative mirror MS/MS spectrum of the peptide ALLENLQEGSVIK from genus *Neisseria*, while Fig. 5C represents the corresponding calibration curve.

Inspired by the significant negative association of these peptides with plasma insulin during OGTT, we explored the possibility that some of these peptides might exhibit incretin-like effects. Previous research has demonstrated the presence of bioactive compounds, such as exendin from the saliva of the Gila monster, which has been shown to have prolonged glucose-lowering effects [64, 65]. Specifically, we performed glucose-induced insulin secretion assay by treating INS-1 *β*-cells with the nine bacterial peptides exhibiting highest significance from the association analysis: AQYLLESLVK, LSNESVQTVR, LLELTPALYVGK, ALLENLQEGSVIK, DNTYATLIVPVGTK, and AVAVVIPTNEELMIAHDTAR from genus *Neisseria* and peptides ALSTTVTGIEMFR, DVSYDEDSITVDGHR, and VYAVEPEASPLLTTGK from genus *Corynebacterium* (Supplementary Fig. 5) compared to vehicle control. The screen revealed that peptides AQYLLESLVK, DNTYATLIVPVGTK, and AVAVVIPTNEELMIAHDTAR from genus *Neisseria* nominally increased insulin secretion under low glucose conditions (Fig. 5D), while *Neisseria* peptides ALLENLQEGSVIK and AVAVVIPTNEELMIAHDTAR nominally increased insulin secretion at high glucose (Fig. 5E) compared to vehicle control. Figure 5F highlights results of *Neisseria* peptide ALLENLQEGSVIK, showing significantly increased insulin secretion at high glucose, but not at low glucose.

## Discussion

Here, we employed an ultrafast state-of-the-art saliva metaproteomics and proteomics workflow to explore differences in salivary microbiome composition and its functions between healthy controls and patients with screen-detected type-2 diabetes. Participants in our study were unaware of their diabetes status until attending the ADDITION-PRO clinical examination. As a result, they had not received any diabetes-specific treatment that could influence our observations. Our comprehensive proteomics and metaproteomics analysis of saliva provided insights at the three different levels — peptide, protein, and genus, each providing complementary information about both human and microbial profile of the studied patient cohort. We identified over 1900 human proteins, more than 2800 bacterial proteins, and 7452 bacterial peptides, attributed to 33 bacterial genera and 7 bacterial phyla. Our results align with previous salivary proteomics studies but stand out by offering comprehensive proteomic depth with significantly shorter measurement times [5, 23, 24, 66]. For instance, the number of proteins identified in a single run is comparable to those reported in study using peptide-level fractionation, likely due to the enhanced sensitivity of modern mass spectrometers [5]. While our rapid metaproteomics analysis does not achieve the comprehensive oral microbiome coverage seen in metagenomic studies of T2D individuals [13, 67–69], it successfully reproduces key earlier observations. Notably, the increased abundance of *Corynebacterium* in T2D individuals is consistent with findings from previous metagenomic studies [69]. The observed proteomics and metaproteomics changes mainly fit into the paradigm of altered immune-, lipid-, and glucose-metabolism regulatory systems, as well as increased oxidative stress and possible precancerous state of T2D oral cavity. In addition, bacterial genera *Neisseria* and *Corynebacterium* show a promising biomarker potential shedding light on disease pathophysiology and potentially opening avenues for microbial interventions in prevention and management of T2D. These findings are further supported by associations with bio-clinical characteristics. We have made efforts to address the possible influence of age, sex, smoking, and BMI by matching participants on these factors. However, the role of additional potential confounders, such as lipid-lowering, anti-inflammatory, and antihypertensive medication as well as differences in lifestyle factors such as diet, physical activity, and oral hygiene, remains to be explored in larger, less-biased populations. Future studies may also elucidate whether the observed differences are likely to be causal by examining prospective associations with diabetes progression or complications development.

T2D is known to disrupt oral homeostasis. For example, T2D conditions can reduce saliva secretion and increase carbohydrate levels in the parotid glands, promoting the growth of specific bacterial species and oral *Candida* [70]. Therefore, it can be hypothesized that T2D influences the composition of the human salivary proteome. In fact, our enrichment analysis of differentially expressed proteins displayed notable shifts in immunity, metabolism, translation/biogenesis, and signaling pathways in T2D individuals. These findings reinforce the role of increased immune activation and metabolic dysregulation in T2D, offering insights into potential disease mechanisms. Additionally, we observed downregulation of keratin types 13, 4, and 78 in T2D saliva, which are established markers of oral cancer [71]. Specifically, reduced expression of keratin types 4 and 13 is associated with oral squamous cell carcinoma and precancerous state/epithelial dysplasia [72, 73]. Decreased levels of keratinocyte sciellin (SCEL) in the T2D group indicate disturbed differentiation of epithelial cells [74]. Additionally, down-regulation of cytoskeletal proteins, including SH3GLB2 (endophilin-B2), spectrin (SPTAN1), and annexin (ANXA2) in T2D individuals mirrors the pattern seen in oral cancers and precancerous lesions [75–79]. Patients with T2D are known to have a higher risk of developing oral cancer and oral potentially malignant disorders (OPMD) compared to nondiabetic individuals [80–82]; therefore, these findings hint at a possible precancerous state of oral cell carcinoma in individuals with T2D. It is noteworthy that among the HC group, there were more former smokers, whereas among the T2D group, there were more current smokers. This suggests that the T2D group likely had a higher cumulative smoking exposure. This factor may significantly contribute to the presence of indicators of oral precancerous changes.

In further characterizing the T2D salivary microbiota, taxonomic assignment of bacterial peptides revealed a diverse landscape. Despite a very short MS measurement time (21 min) per sample, our results align with prior salivary metaproteomics studies that employed longer measurement time, particularly in terms of bacterial taxonomic abundance and relative composition of human saliva [4, 57, 78]. The bacterial signature obtained from 16S rRNA data and metaproteomics analysis was vastly similar. However, slight discrepancies in abundance of the top20 bacterial genera, particularly genus *Corynebacterium*, may stem from amplification-related variability inherent to 16S rRNA sequencing [83], highlighting the complementary nature of these approaches. Our identification of bacterial genera *Corynebacterium*, *Neisseria*, and *Haemophilus*, as potential salivary biomarkers of T2D, aligns with previous literature linking these genera to metabolic syndrome, compromised immunity, and diabetes-related complications [56, 84–87]. For instance, an increased abundance of genus *Corynebacterium* has been previously observed in the patients with metabolic syndrome, where it displayed significant correlation with fasting blood glucose levels [84]. Moreover, genus *Corynebacterium* is known to colonize individuals with compromised immune system [85]. This aligns with our findings of enriched immunological processes, alongside upregulation of immunoglobulins and complement factors, in T2D patients. Furthermore, bacterial species *Corynebacterium matruchotii* and *Neisseria mucosa* have been previously linked to mucosal inflammation and increased salivary glucose levels [86, 87]. Finally, the genus *Haemophilus* has been suggested as a microbial biomarker in Chinese patients with type-2 diabetes [56]. These findings underscore the biomarker potential of these bacterial genera and their relevance in understanding T2D pathophysiology. Nevertheless, since these bacterial genera include multiple species, some of which are considered beneficial, while the others pathogenic, further studies should focus on species/strain level identification, as this taxonomic resolution is needed to fully understand the role of individual species/strains in the pathophysiology of T2D.

Functional enrichment analysis of bacterial peptides unveiled oxidative stress-related terms, indicative of the potential role of microbial factors in exacerbating oxidative stress in T2D. Human peroxidases play a crucial role in innate immunity, apoptosis, and cell signaling. Excessive peroxidase activity can lead to oxidative damage in cells and tissues, contributing to various diseases [88]. Oxidative stress, mediated by reactive oxygen species (ROS), is linked to the development of insulin resistance, *β*-cell dysfunction, impaired glucose tolerance, type-2 diabetes, and diabetes complications [89]. Bacterial peroxidases have also been implicated in degrading phenolic compounds [90, 91], potentially exacerbating oxidative damage. Based on this, we can assume that bacterial redox machinery plays a role in the pathophysiology of T2D. We conducted enrichment analysis on unique bacterial peptides from genera *Neisseria* and *Corynebacterium*, which showed significant differences in abundance between the two patient groups. Specifically, glycolysis-related functions were enriched for the peptides from genus *Corynebacterium* including those involved in NAD( +) or NADP( +) binding and glyceraldehyde 3-phosphate dehydrogenase type I activity. Interestingly, an increase in the population of *Corynebacterium glutamicum* has been previously linked to improved glucose metabolism in T2D mice [92]. Furthermore, previous studies have linked increased abundance of the genus *Corynebacterium* to high blood glucose levels in patients with metabolic syndrome [84]. Additionally, given its association with colonization of individuals with weakened immune system [85], we hypothesize that genus *Corynebacterium* may exhibit opportunistic behavior while providing protection against T2D and its complications.

Moreover, our association analysis revealed clinically relevant links between bacterial peptides and cardiometabolic traits, suggesting a complex interplay between specific bacterial genera and insulin secretion. In vitro validation of these peptides supported effects on insulin secretion of some of these individual peptides, further suggesting their potential relevance in T2D pathophysiology. In particular, peptide ALLENLQEGSVIK from genus *Neisseria* emerged as a promising candidate, increasing glucose-stimulated insulin secretion (~ 25%), which aligns with our clinical association results. To contenxtualize these results, the incretin GLP-1 and its analogues increase glucose-stimulated insulin secretion 2-3 fold in INS-1 cells [93–95]. While these findings provide intriguing insights into the potential impact of bacterial peptides on insulin secretion, it is important to stress that their combined effects were not tested, and that the abovementioned identifications were carried out in saliva samples, thus leaving unclear the mechanism of their potential influence on host insulin secretion by pancreatic *β*-cells.

Overall, our study provides a comprehensive overview of the T2D salivary microbiome and its intricate relationship with host proteomics and metabolic regulation. Leveraging the power of DIA-PASEF methodology, our study offers valuable insights into T2D pathophysiology and highlights the promise of metaproteomics in clinical research. Nevertheless, the limitation of this study, which includes small sample size, cross-sectional study design, underscores the need for further validation of our findings in larger and prospective cohorts of individuals with diabetes and elevated diabetes risk.

## Conclusions

In conclusion, our results showed significant salivary metaproteome differences between T2D patients and healthy controls and discovered disease-specific bacterial genera, which possess biomarker potential. Our findings offer solid evidence of altered immune-, lipid-, and glucose-metabolism regulatory systems, as well as increased oxidative stress and possible precancerous changes in T2D saliva. This metaproteomics dataset can serve as a valuable resource for the scientific community.

## Supplementary Information

Additional file 1. Total number of identified proteins (human + bacteria) in saliva samples.

Additional file 2. Differentially expressed proteins between type-2-diabetic patients and healthy controls.

Additional file 3. 1D functional enrichment analysis of differentially expressed proteins.

Additional file 4. Total number of identified bacterial genera and phyla, using orthogonal sequencing techniques.

Additional file 5. 2D functional enrichment analysis of all peptides between the two patient groups.

Additional file 6. Results of clinical association analysis between bacterial peptides of interest and patient cardiometabolic traits.

Additional file 7. Calculation of bacterial peptide concentrations in saliva samples, using synthetic peptide hybrid library.

Additional file 8. Results of interaction analysis between bacterial peptides of interest and patient cardiometabolic traits.

Additional file 9: Supplementary Figure 1. Principal component analysis of salivary proteins. A) Percentage of explained variance, calculated for the 10 principal components of principal component analysis. X-axis depicts the component (PC1-PC10), while y-axis shows the corresponding % of variance, explained by each component; B) Top25 separation drivers for principal components PC1 and PC2. X-axis depicts % of contribution, while y-axis - the corresponding protein-coding gene names.

Additional file 10: Supplementary Figure 2. Correlation of bacterial genera between the two datasets (metaproteomics and 16S rRNA). Scatter plot, demonstrating correlation between genus abundances, detected by the two sequencing methodologies (metaproteomics vs 16S rRNA sequencing), where MS on the y-axis stands for metaproteomics and 16S rRNA on the x-axis – for 16S rRNA sequencing. The correlation was estimated, using statistically-significant Pearson correlation coefficient.

Additional file 11: Supplementary Figure 3. Extended version of the clinical association analysis. Clinical association analysis of all bacterial peptides from genera *Corynebacterium*, *Haemophilus* and *Neisseria*, arranged as a supervised hierarchical clustering and visualized, as a heatmap, based on scaled log2-transformed peptide intensities. Colors indicate specific classes of cardiometabolic traits used in association analysis, as well as bacterial genera.

Additional file 12: Supplementary Figure 4. Annotated mirror MS/MS spectra for validation of bacterial peptides. Annotated mirror MS/MS spectra, used for verification of peptide identity by synthetic peptide hybrid library search. The spectra of the six bacterial peptides from genera *Neisseria* and *Corynebacterium*, validated as a result of above mentioned analysis, are included.

Additional file 13: Supplementary Figure 5. Normalized insulin secretion of bacterial peptides. Screen of nine bacterial peptides on insulin secretion at low glucose (baseline, 2 mM glucose) and high glucose (20 mM) compared to vehicle control in INS-1 β-cell line. Bar plots depicting absolute values (normalized to protein content) of the baseline (A) and high-glucose (B) insulin secretion for each of the screened bacterial peptides. C) Difference in stimulation index (ratio of insulin secretion at high glucose to insulin secretion at low glucose) of all nine bacterial peptides compared to vehicle control. Peptides AQYLLESLVK, LLELTPALYVGK and DNTYATLIVPVGTK (genus *Neisseria*) nominally decreased stimulation index. Data was screened with multiple unpaired t-tests and depicted as % difference from vehicle control values of individual batches (batches indicated by symbols). D) Stimulation index (absolute and % change from vehicle control) of peptide ALLENLQEGSVIK compared to vehicle control. Data was tested with unpaired t-test (batches indicated by symbols).

## Data Availability

The mass spectrometry proteomics data have been deposited to the ProteomeXchange Consortium via the PRIDE partner repository with the dataset identifier PXD051453.

## Abbreviations

ACN: Acetonitrile
BMI: Body mass index
DIA: Data-independent acquisition
FA: Formic acid
FDR: False discovery rate
FLC: Free light chain
GAPDH: Glyceraldehyde 3-phosphate dehydrogenase
HC: Healthy control
LC/MS: Liquid chromatography/mass spectrometry
LC–MS/MS: Liquid chromatography/tandem mass spectrometry
MS: Mass spectrometry
OGTT: Oral glucose tolerance test
OPMD: Oral potentially malignant disorders
PCA: Principal component analysis
ROS: Reactive oxygen species
SD: Standard deviation
T2D: Type-2 diabetes
TFA: Trifluoroacetic acid
16S rRNA: 16S ribosomal ribonucleic acid sequencing
WHO: World Health Organization
