# Supplementary figures and images for: Salivary proteomics and metaproteomics identifies distinct molecular and taxonomic signatures of type-2 diabetes
Source: Microbiome. 2025 Jan 10;13:5. doi: 10.1186/s40168-024-01997-5 (PMC11720885; doi:10.1186/s40168-024-01997-5)

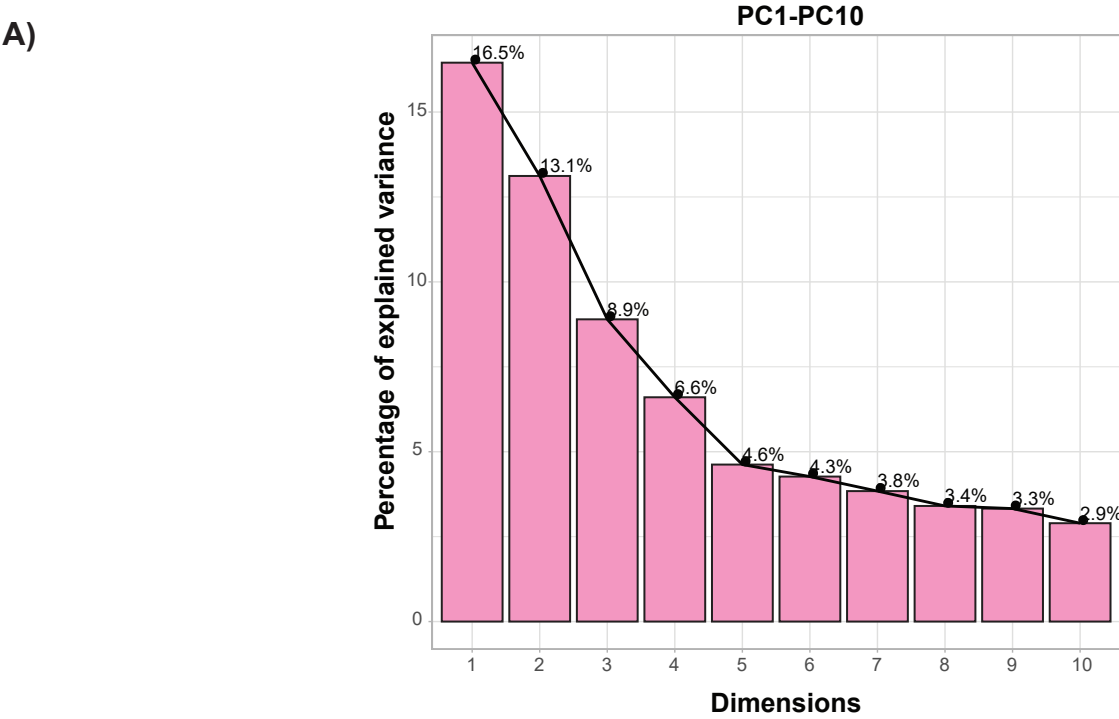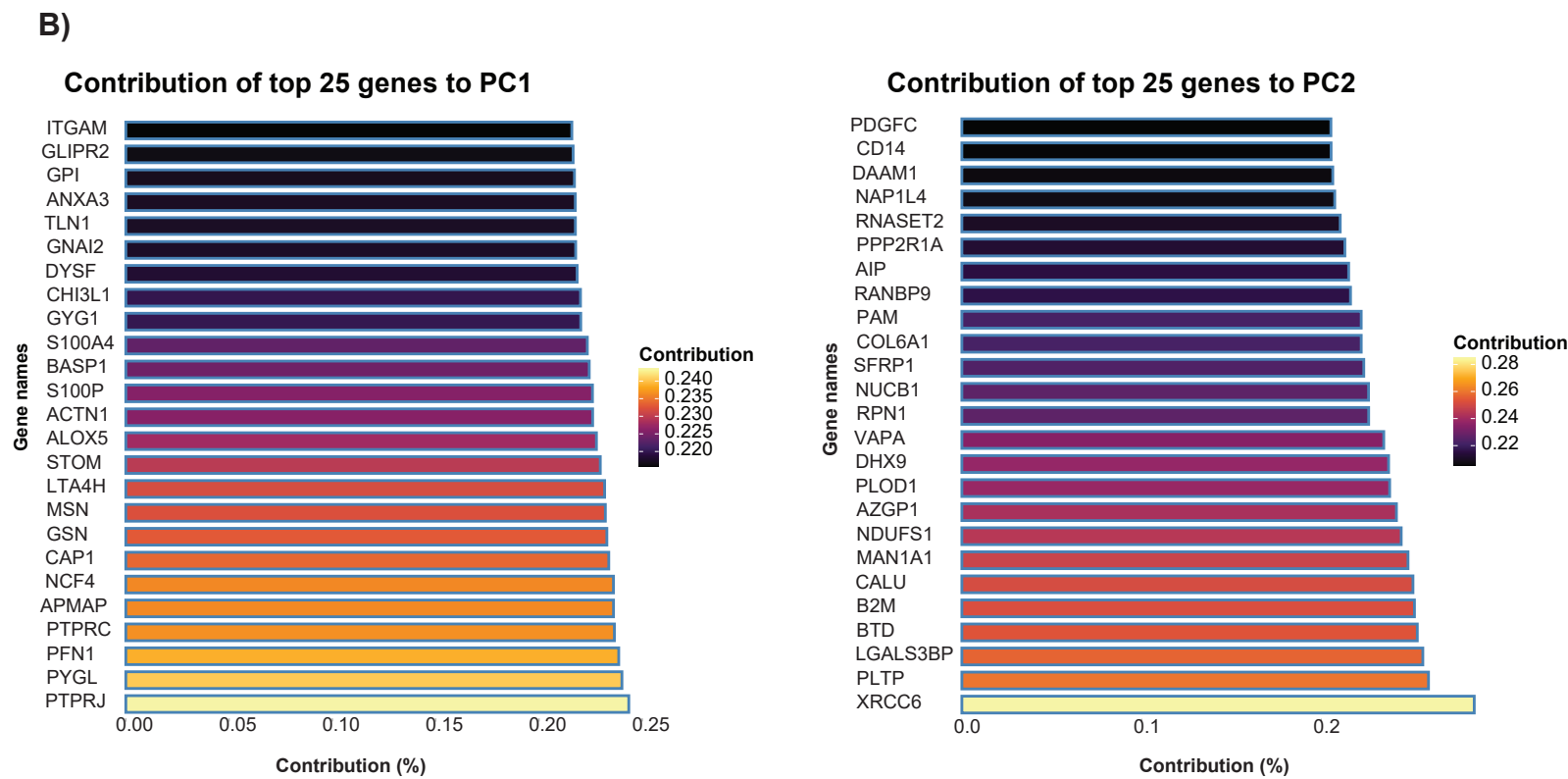

Supplementary Figure 1

Supplement: Supplementary file 10 — Additional file 9: Supplementary Figure 1. Principal component analysis of salivary proteins. A) Percentage of explained variance, calculated for the 10 principal components of principal component analysis. X-axis depicts the component (PC1-PC10), while y-axis shows the corresponding % of variance, explained by each component; B) Top25 separation drivers for principal components PC1 and PC2. X-axis depicts % of contribution, while y-axis - the corresponding protein-coding gene names. [file 40168_2024_1997_MOESM9_ESM.pdf]

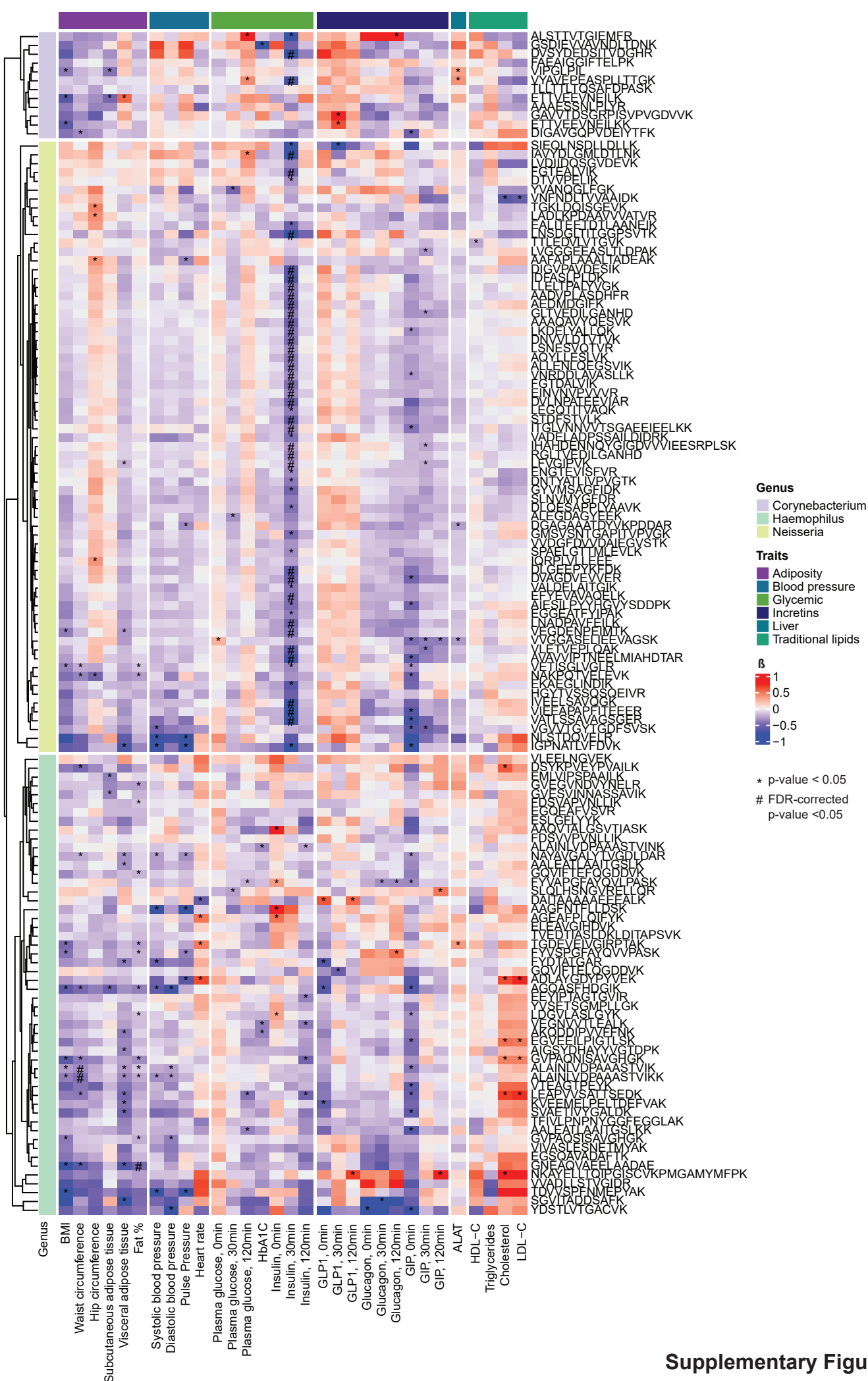

Supplementary Figure 3

Supplement: Supplementary file 12 — Additional file 11: Supplementary Figure 3. Extended version of the clinical association analysis. Clinical association analysis of all bacterial peptides from genera Corynebacterium, Haemophilus and Neisseria, arranged as a supervised hierarchical clustering and visualized, as a heatmap, based on scaled log2-transformed peptide intensities. Colors indicate specific classes of cardiometabolic traits used in association analysis, as well as bacterial genera. [file 40168_2024_1997_MOESM11_ESM.pdf]

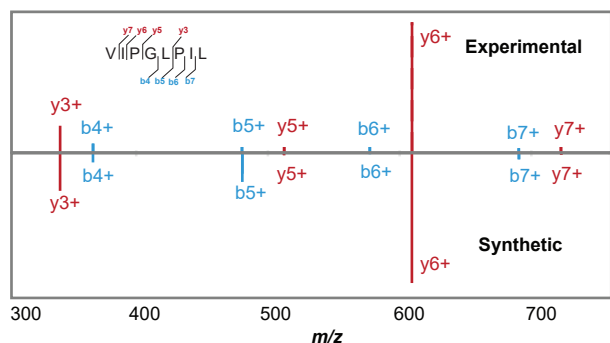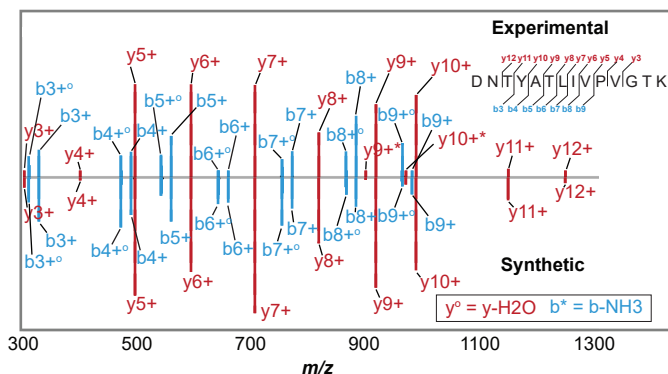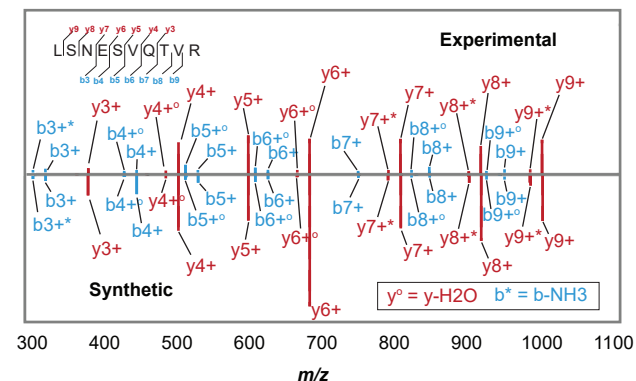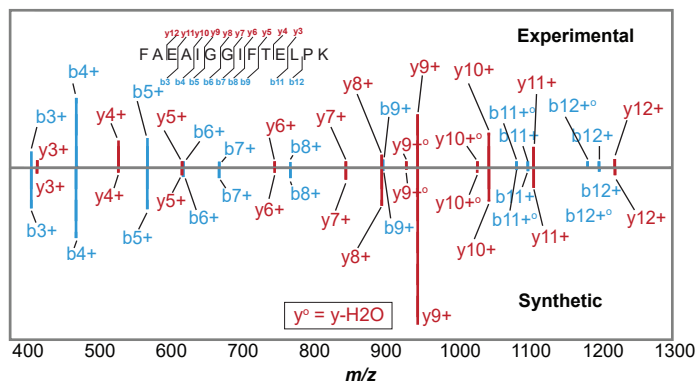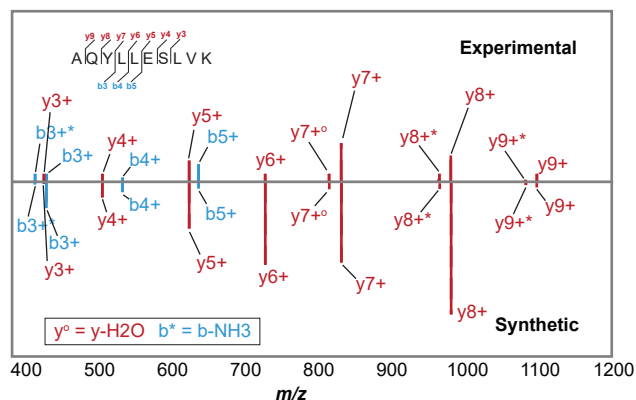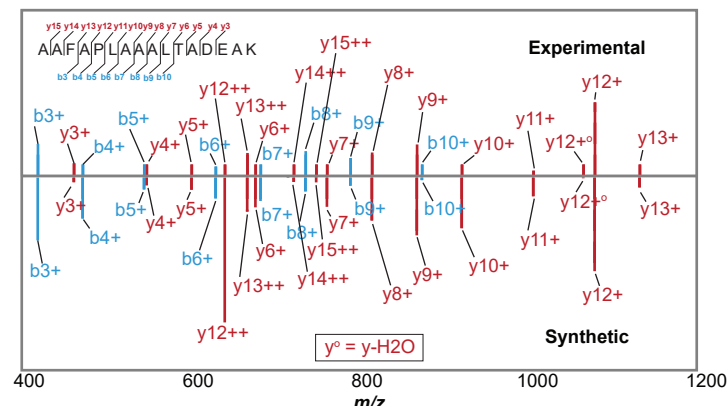

Supplement: Supplementary file 13 — Additional file 12: Supplementary Figure 4. Annotated mirror MS/MS spectra for validation of bacterial peptides. Annotated mirror MS/MS spectra, used for verification of peptide identity by synthetic peptide hybrid library search. The spectra of the six bacterial peptides from genera Neisseria and Corynebacterium, validated as a result of above mentioned analysis, are included. [file 40168_2024_1997_MOESM12_ESM.pdf]

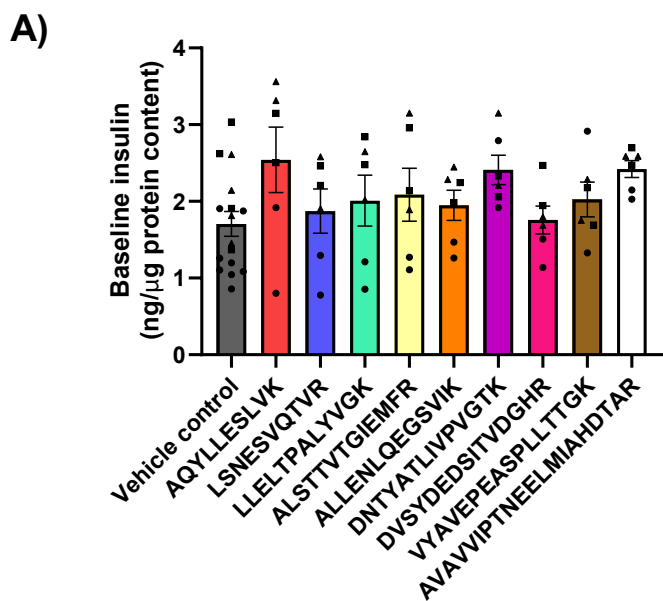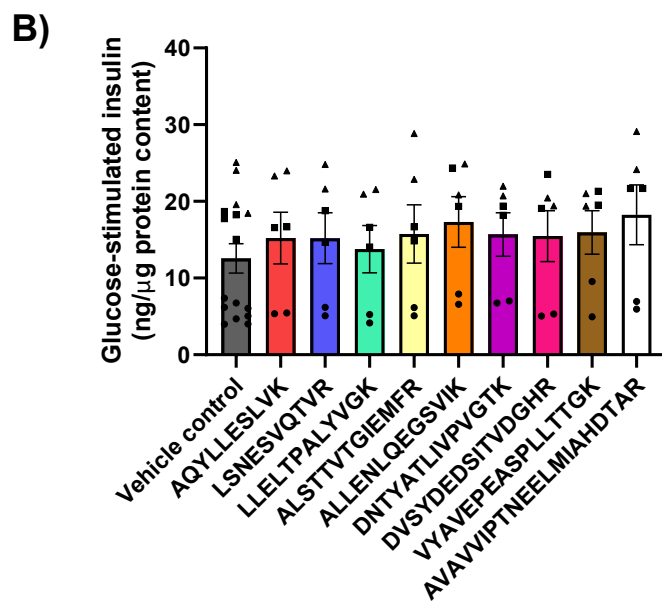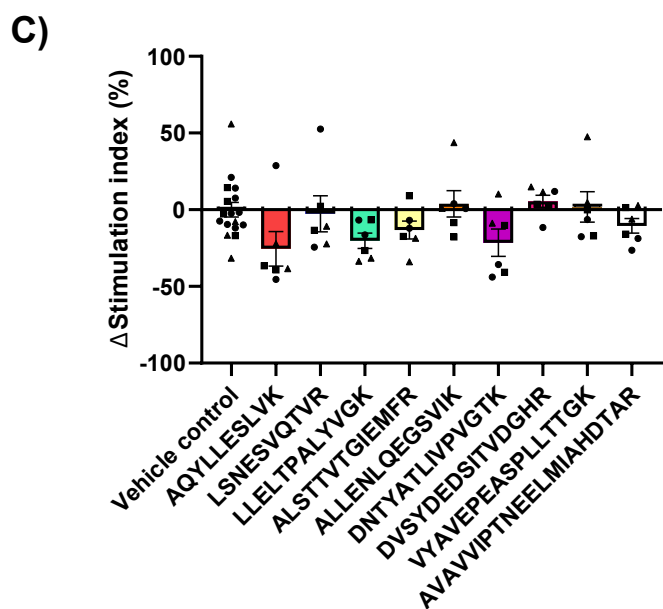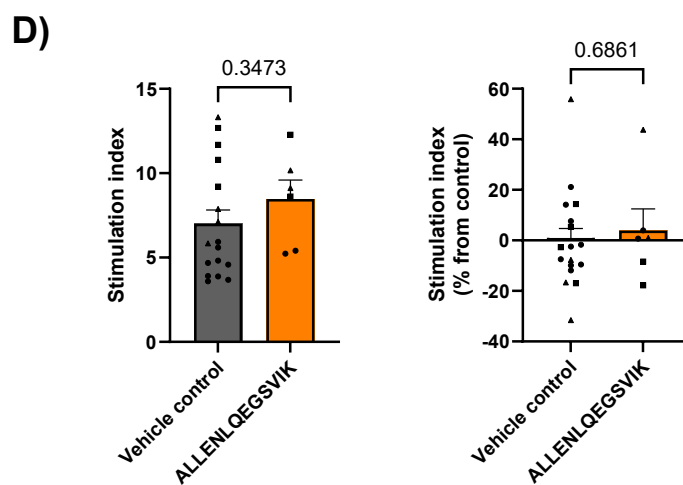

Supplement: Supplementary file 14 — Additional file 13: Supplementary Figure 5. Normalized insulin secretion of bacterial peptides. Screen of nine bacterial peptides on insulin secretion at low glucose (baseline, 2 mM glucose) and high glucose (20 mM) compared to vehicle control in INS-1 β-cell line. Bar plots depicting absolute values (normalized to protein content) of the baseline (A) and high-glucose (B) insulin secretion for each of the screened bacterial peptides. C) Difference in stimulation index (ratio of insulin secretion at high glucose to insulin secretion at low glucose) of all nine bacterial peptides compared to vehicle control. Peptides AQYLLESLVK, LLELTPALYVGK and DNTYATLIVPVGTK (genus Neisseria) nominally decreased stimulation index. Data was screened with multiple unpaired t-tests and depicted as % difference from vehicle control values of individual batches (batches indicated by symbols). D) Stimulation index (absolute and % change from vehicle control) of peptide ALLENLQEGSVIK compared to vehicle control. Data was tested with unpaired t-test (batches indicated by symbols). [file 40168_2024_1997_MOESM13_ESM.pdf]
