# Supplementary material for: Salivary proteomics and metaproteomics identifies distinct molecular and taxonomic signatures of type-2 diabetes
Source: Microbiome. 2025 Jan 10;13:5. doi: 10.1186/s40168-024-01997-5 (PMC11720885; doi:10.1186/s40168-024-01997-5)
Supplement: Supplementary file 11 — Additional file 10: Supplementary Figure 2. Correlation of bacterial genera between the two datasets (metaproteomics and 16S rRNA). Scatter plot, demonstrating correlation between genus abundances, detected by the two sequencing methodologies (metaproteomics vs 16S rRNA sequencing), where MS on the y-axis stands for metaproteomics and 16S rRNA on the x-axis – for 16S rRNA sequencing. The correlation was estimated, using statistically-significant Pearson correlation coefficient. [file 40168_2024_1997_MOESM10_ESM.pdf]

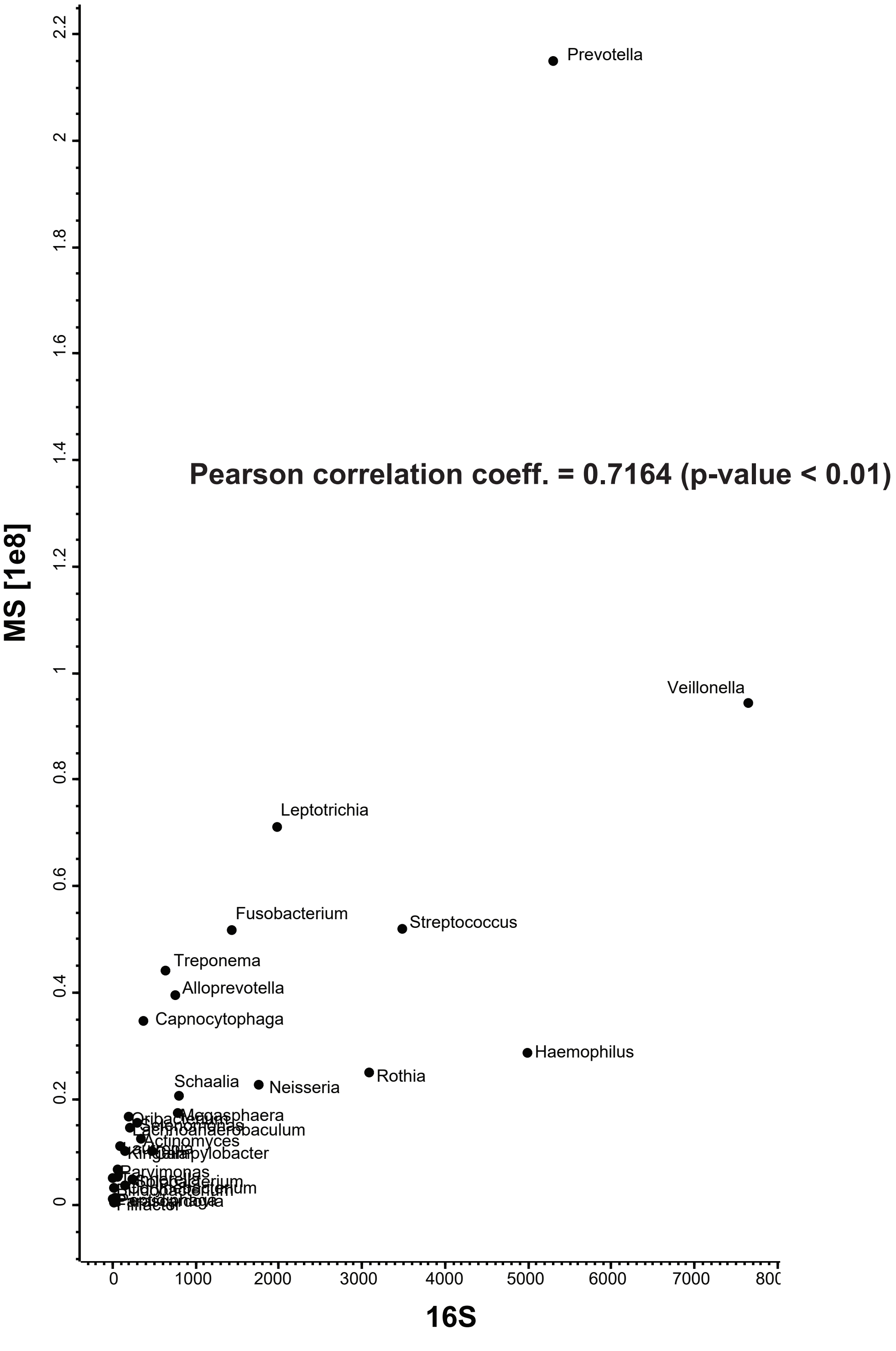

Supplementary Figure 2
